# Mild encephalitis/encephalopathy with a reversible splenial lesion secondary to encephalitis complicated by hyponatremia: A case report and literature review

**DOI:** 10.1097/MD.0000000000017982

**Published:** 2019-11-22

**Authors:** Bi-chuan Shi, Jiao Li, Ji-wei Jiang, Mei-xin Li, Jian Zhang, Xiu-li Shang

**Affiliations:** Department of Neurology, The First Affiliated Hospital of China Medical University, Shenyang, Liaoning Province, China.

**Keywords:** encephalitis, hyponatremia, interleukin-6, mild encephalitis/encephalopathy with a reversible splenial lesion

## Abstract

**Rationale:**

Mild encephalitis/encephalopathy with a reversible splenial lesion (MERS) is an infection-associated encephalitis/encephalopathy syndrome that is predominately caused by a virus. MERS has no direct association with central nervous system (CNS) infections or inflammation. Non-CNS infections may cause reversible lesion in the splenium of corpus callosum. Recently, there have been reports of many patients with hyponatremia related MERS. Interleukin-6 (IL-6) was also found elevated in serum and in cerebrospinal fluid (CSF) in patients with MERS. The role of IL-6 in the non-osmotic release of vasopressin is crucial. Persistent hyponatremia may be linked to this effect. The following is a case report of MERS secondary to encephalitis, complicated by hyponatremia. We will summarize the latest research and progress regarding MERS.

**Patient concerns:**

A 31-year-old man was admitted to our department with a 5-day history of fever and headache. His initial diagnosis was encephalitis and hyponatremia; during this period the patient also developed MERS secondary to the encephalitis.

**Diagnoses:**

Encephalitis was diagnosed by reviewing the history of fever, headache, neck rigidity and Kerning sign (+) on clinical examination. Lab tests revealed: serum VCA IgG (+), EBNA-1 IgG (−), EBV IgM (−), and inflammation in the analysis of CSF. Cranial MRI+C showed that the blood vessels on the surface of the brain were obviously increasing and thickening and diffuse slow waves were detected on the electroencephalogram (EEG). The patient's hyponatremia aggravated on the third day of hospitalization. On the fourth day of hospitalization, the patient was somnolent, apathetic, and slow. Magnetic resonance imaging (MRI) of the brain, with a T2-weighted fluid attenuated inversion recovery image, showed high-signal intensity in the splenium of the corpus callosum (SCC) on the fifth day of hospitalization. Diffusion-weighted imaging (DWI) showed splenial hyperintensity as a “boomerang sign” and reduced diffusion on apparent diffusion coefficient (ADC) maps. Cranial MRI findings returned to normal after 1 month. The diagnosis of MERS was confirmed.

**Interventions:**

We administered an intravenous drip infusion of acyclovir and prescribed oral sodium supplementation.

**Outcomes:**

The patient's neurological symptoms gradually improved. The MRI lesion in the SCC disappeared on the 30th day.

**Lessons:**

In patients with encephalitis accompanied by hyponatremia, elevated IL-6 or urinary β2-microglobulin (β2MG), and exacerbations such as sudden somnolence, delirium, confusion, and seizures, the possibility of secondary MERS should be investigated, in addition to the progression of encephalitis.

## 1 Introduction

Reversible splenial lesion syndrome (RESLES) was first identified by Carlos Garcia-Monco^[1]^ in 2011. It is a rare clinic-radiological entity with an excellent prognosis. Clinical presentation is nonspecific; however, use of magnetic resonance imaging (MRI) can detect a reversible lesion in the splenium of corpus callosum. Several disorders of varied origin are associated with it, including infection, high-altitude cerebral edema, seizures, antiepileptic drug (AED) withdrawal, and metabolic disturbances.^[1]^ Mild encephalitis/encephalopathy with a reversible splenial lesion (MERS),^[2]^ is defined as an acute encephalitis/encephalopathy caused by an acute inflammatory disease. Compared with RESLES which is caused by other disorders, MERS is only secondary to an infection such as influenza virus, rotavirus, and O-157 *Escherichia coli*, etc, and it's clinical manifestations are similar to encephalitis.^[1]^ Broadly speaking, the spectrum of RESLES includes specific diseases, such as clinically MERS and Marchiafava-Bignami disease.^[3]^

Recently, many patients with MERS associated with hyponatremia have been reported. In order to better understand this phenomenon, Takanashi et al,^[4]^ evaluated sodium (Na) levels in a series of patients with MERS. These values compared with those of age-matched patients with mild upper respiratory infections, values of patients with other types of encephalopathy, and values of patients with febrile seizures. There were significant differences between the Na levels of patients with MERS and those in other groups (Na levels are decreased in patients with MERS). Regarding pathophysiology, it is necessary to consider further studies of urine, blood osmolality, and antidiuretic hormone in patients with MERS.

Currently, there are fewer cases of viral encephalitis complicated with MERS. This case report concerned a 31-year-old patient with MERS secondary to encephalitis and hyponatremia. We will summarize and conclude the latest progress of MERS research in order to better understand this syndrome.

## 2 Case report

**Figure 1:**
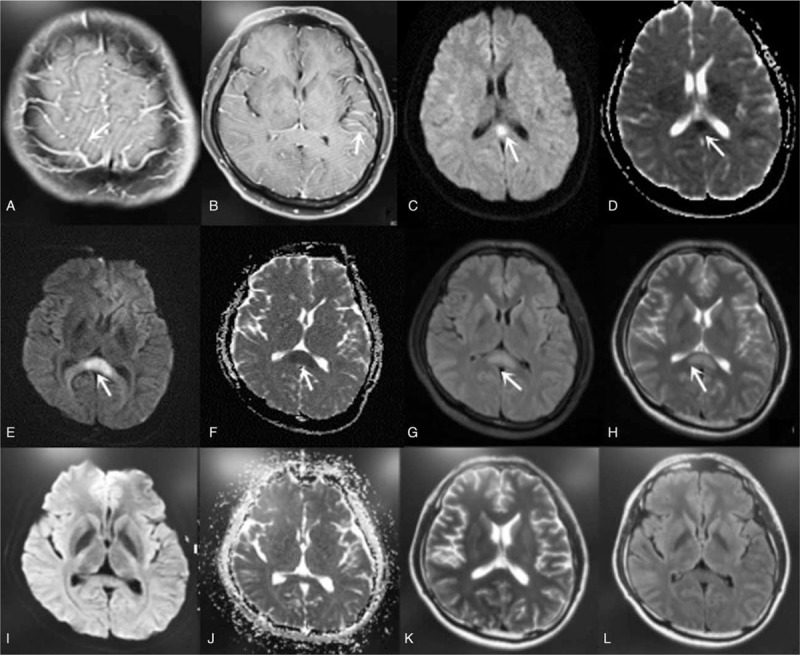
MRI+C (A, B) showed the blood vessels on the surface of the brain are obviously increased and strengthened. On day 4, DWI (C) showed high signal intensity and ADC (D) low signal intensity in the splenium of corpus callosum. On the 5th day, MRI showed FLAIR (G) and T2 (H) high signal in the splenium of corpus callosum, DWI (E) showed splenial hyperintensity as a “boomerang sign” and reduced diffusion on ADC (F) maps. Cranial MRI returned to normal on day 30 (I–L). ADC = apparent diffusion coefficient; MRI = magnetic resonance imaging.

In July 9, 2018, a previously healthy 31-year-old man was admitted to our hospital due to a 5-day history of fever (up to 39.5 °C) and headache, accompanied by nausea, and vomiting. Initial examination revealed cervical lymphadenopathy, neck rigidity, Kerning sign (+), and an otherwise normal neurological examination. Laboratory results revealed hyponatremia (sodium, 135 mmol/L), a serum white blood cell (WBC) count of 10.23 × 109/L (monocytes 11%), C-reactive protein (CRP) of 9.50 mg/L, serum EBV antibodies showed VCA IgG (+), EBNA-1 IgG (–), and EBV IgM (–). A lumbar puncture indicated an elevated cerebrospinal fluid (CSF) pressure of 180 mmH_2_O, a WBC count of 121 × 106/L, elevation of total proteins (1762 mg/L) and glucose was 2.6 mmol/L. Serum and CSF were negative for NMDARAb, AMPA1Ab, AMPA2Ab, LG1Ab, CASPR2Ab, GABABRAb. An electroencephalogram (EEG) revealed diffuse slow waves. Cranial MRI+C showed the blood vessels on the surface of the brain were obviously increasing and thickening (Fig. 1). In summary, encephalitis was considered. Epstein–Barr virus was suspected. VCA IgG can be present without VCA IgM or EBNA-1 IgG in case of acute or past infection. Antiviral treatment began on the day of diagnosis, consisting of acyclovir (500 mg every 8 hours) for 2 weeks and mannitol (250 mL every 8 hours), and sodium supplementation was also initiated (10% sodium chloride, 20 mL).

Hyponatremia (sodium, 130 mmol/L) was persistent on the third day of hospitalization, despite sodium supplementation. On the fourth day of admission, he showed a somnolent, apathetic, and slow response. DWI showed high signal intensity and ADC low signal intensity in the splenium of corpus callosum (Fig. 1). The patient continued to receive acyclovir intravenous drip infusion (IV) and sodium supplementation (10% sodium chloride, 40 mL). On the fifth day of hospitalization, his temperature returned to normal. MRI of the brain showed high-signal intensity in the splenium of corpus callosum (SCC) on a T2-weighted fluid attenuated inversion recovery image. Diffusion-weighted imaging displayed splenial hyperintensity as a “boomerang sign”^[5]^ and reduced diffusion on apparent diffusion coefficient (ADC) maps (Fig. 1).

Consciousness improved on the seventh day. On the 15th day after admission, headache, vomiting, and neck rigidity gradually disappeared. The patient was discharged on the 17th day. Cerebrospinal fluid (CSF) examination showed that CSF parameters had returned to normal and there was no evidence of hyponatremia (sodium, 137.6 mmol/L). On the 30th day, a cranial MRI showed that lesions in the SCC had almost disappeared (Fig. 1), and the diagnosis of MERS was confirmed.

This study was approved by the Ethics Committee of the First Affiliated Hospital of China Medical University and adhered to the tenets of the Declaration of Helsinki. Informed written consent was obtained from the patient for publication of this case report and the accompanying images.

## 3 Literature review

To summarize the latest developments of MERS, we used the following terms for a PubMed search: “reversible” and “Splenial” and “lesion,” “MERS,” “RESLES” for the literature review. From 2016 to 2018, a total of 32 MERS patients (case reports and other original studies/case series) were contained in the literature.

### 3.1 Data

A total of 32 cases meeting the MERS criteria were included^[6–28]^ (Tables 1–3). Most patients were children or young (median age 16 years, range 2–59 years), men were more common (17 men, 15 women).

**Table 1:**
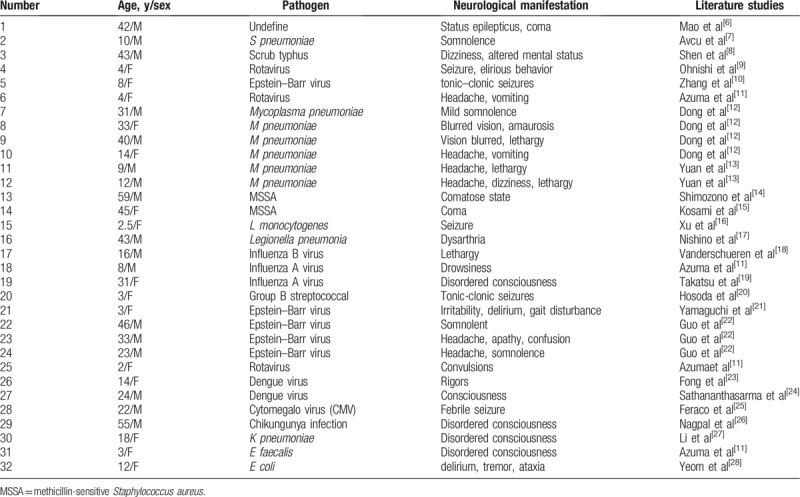
Age, sex, pathogen, and neurological manifestation of MERS cases 2016 to 2018.

**Table 2:**
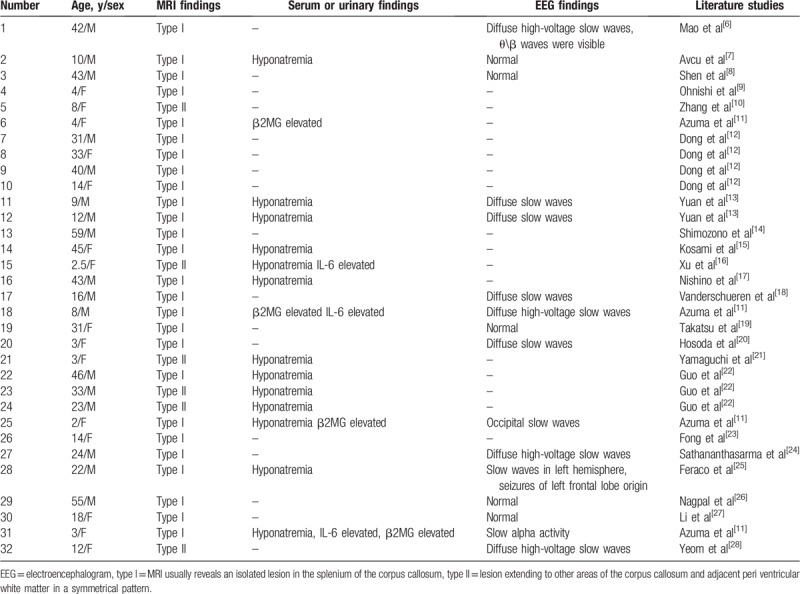
MRI findings, serum or urinary findings, and EEG findings of MERS cases 2016–2018.

**Table 3:**
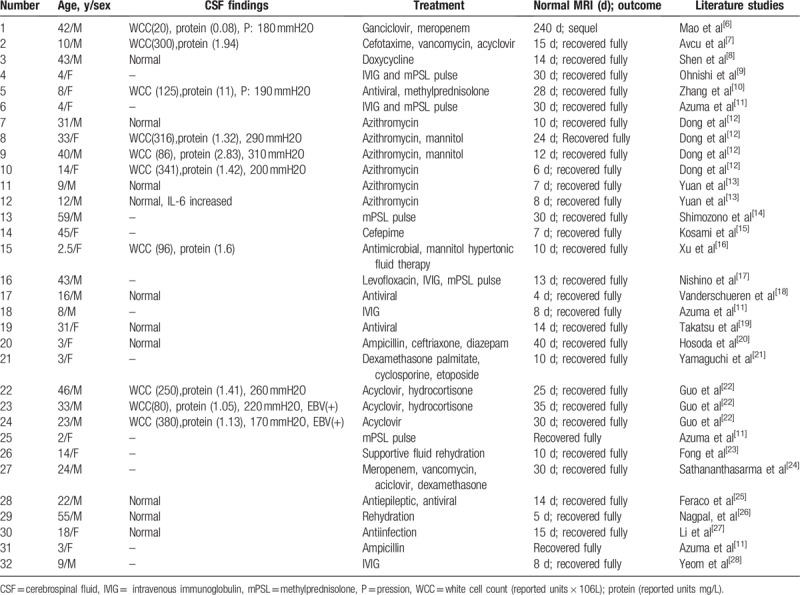
CSF findings, treatment, normal MRI(d), outcome of MERS cases 2016–2018.

### 3.2 Pathogen

MERS is an encephalitis/encephalopathy syndrome associated with infection, Carlos Garcia-Monco^[1]^ found that the dominant cause is a virus, especially the influenza virus. There are other pathogens, which include rotavirus, measles, herpes virus 6, Epstein-Barr virus, chickenpox-herpes zoster virus, mumps, adenovirus, *Salmonella enteritis*, *E coli*, and *Legionella pneumophila*. In recent studies (Table 1), additional bacteria and rare viruses have been reported: Mycoplasma pneumoniae (n = 6), methicillin-sensitive *Staphylococcus aureus* (MSSA) (n = 2), and dengue virus (n = 2). The following pathogens have also been identified: *Klebsiella pneumoniae*, *Enterococcus faecalis*, *Streptococcus pneumoniae*, scrub typhus, *Listeria monocytogenes*, Chikungunya, and Group B Streptococcus, etc. These have all greatly enriched the pathogen spectrum of MERS.

### 3.3 Clinical manifestations

Prodromal manifestations were as follows: fever, nausea, vomiting, cough, rash, and joint pain. Symptoms of neurological manifestations included headache, somnolence, delirium, confusion, seizures, ataxia, dysarthria, and blurred vision. Physical examination included an evaluation for neck stiffness and Kernig sign (+).

### 3.4 Serum or urinary findings

Hyponatremia was present in 13 out of 32 patients. In 3 patients IL-6 was elevated (patients 15, 18, and 31) and in 4 patients urinary β2MG was elevated (patients 6, 18, 25, and 31) as shown in Table 2. It is postulated that the increased levels of pre-inflammatory cytokines such as IL-6 may play a significant role in the pathogenesis of the lesion.^[29]^ An acute surge in cytokine levels in the blood stream and CSF cavity may trigger vasodilatation following a reversible vasogenic edema of the myelin. In addition, persistent hyponatremia (not responsive to sodium supplementation) may be due to inappropriate antidiuretic hormone secretion. A growing body of evidence now refers to an important role for interleukin-6 (IL-6) in the non-osmotic release of vasopressin (antidiuretic hormone).^[30]^ As for elevated β2MG, we propose that elevated urinary β2MG levels were not indicative of proximal tubule dysfunction (absence of proximal tubule dysfunction), but were reflective of an excessive immune response. This may represent the underlying cause of reversible SCC lesions.^[13]^ IL-6 and β2MG may be nonspecific but may be relatively sensitive markers. However, only a few cases have been evaluated for serum/cerebrospinal fluid cytokines and β2MG. Further studies are required in order to clarify the relationship of hyponatremia, IL-6, β2MG, and reversible SCC lesions.

### 3.5 MRI findings

Mild encephalitis/encephalopathy with a reversible splenial lesion, displayed T2, DWI hyperintensity with corresponding reduced diffusion on ADC maps, T1 hypo-isointensity, and no contrast enhancement. The lesion shown on MRI could completely disappear on follow-up. During the acute episode, the MRI usually reveals an isolated lesion in the splenium of the corpus callosum (SCC), this is classified as MERS type I, sometimes also extending to other areas of the corpus callosum and adjacent peri ventricular white matter in a symmetrical pattern, this is classified as MERS type II.^[31]^ Our data was MERS type I (81.25%) and MERS type II (18.75%) shown in Table 2.

### 3.6 EEG examinations

An initial EEG examination was conducted in 16 out of 32 cases, 11 of which showed abnormalities, such as diffuse slow waves, occipital slow wave, and diffuse high-voltage slow waves. One patient (patient 28) had secondary generalized seizures originating in the left frontal lobe (Table 2). Oguri et al^[32]^ reported that the MERS group showed a significantly higher theta band power in the frontal and center of the parietal lobes. In previous cases, the frequency of the occurrence of theta waves was not investigated. This may be used as a basis for diagnosis of MERS in the future.

### 3.7 CSF findings

The diagnosis of encephalitis was defined as acute onset of brain dysfunction with CSF pleocytosis. When there was clinical evidence of diffuse brain dysfunction, without evidence of inflammatory changes in the CSF, the term encephalopathy was used. Our cases showed that 20 out of 32 patients had cerebrospinal fluid examinations, 50% were encephalitis (we found that EBV (+) in patients 23 and 24) and 50% were encephalopathy (patient 12, we found that IL-6 was elevated in CSF) shown in Table 3.

### 3.8 Therapy and prognosis

After anti-bacterial, anti-viral, and immunosuppressive treatments, most patients recover within 1 month, without sequelae (Table 3). However, Mao et al^[6]^ reported one severe encephalitis/encephalopathy with reversible splenial lesion of the corpus callosum, that presented with symptoms that included persistent fever, progressive disturbance of consciousness, status epilepticus, and generalized tonic clonic seizures. These symptoms lasted 2 weeks, and the memory impairment persisted at an 8-month follow-up. Thus, the clinical symptom spectrum of MERS requires further research because the clinical symptoms can be severe. Clinicians should be alert for the potential neurological sequela. In addition, Zhang et al^[3]^ indicated that the prognosis of reversible splenial lesion syndrome, characterized by severe, acute to subacute disturbance of consciousness, particularly with extracallosal lesions and diffuse slow waves on the EEG, is poor and includes considerable disability.

## 4 Discussion

MERS is an infection-associated encephalopathy syndrome rather than encephalitis.^[33]^ The pathophysiology of MERS, especially the selective splenial lesions in SCC, is not well understood. Several hypotheses for the mechanism of MERS include intramyelinic axonal edema,^[2]^ inflammatory infiltrates,^[2]^ immune system activation and oxidative stress,^[34]^ as well as fluid imbalance.^[4]^ In the intramyelinic axonal edema hypothesis, the edema and diffusion restriction in the reversible lesions in SCC has been attributed to excitotoxicity, which is due to increased glutamate in the extracellular space. Glutamate binding to non-N-methyl-D-aspartate receptors induces sodium entry, and therefore, cytotoxic edema. If glial cell myelination occurs, intramedullary edema forms. However, a neonate^[35]^ showing an identical lesion in SCC with incomplete myelination did not confirm the intramyelinic edema hypothesis. In the inflammatory infiltrate hypothesis, the influx of inflammatory cells and macromolecules, combined with cytotoxic edema, may result in a decrease in ADC. If the cause resolves quickly, ADC may return to normal. However, almost half of the reported patients had systemic infection encephalopathy, with normal CSF findings. In the immune system activation, oxidative stress, and fluid imbalance hypotheses, MERS had no direct association with CNS infection or inflammation; non-CNS infections may cause MERS in SCC.

In recent cases, elevated levels of IL-6 in serum and CSF were detected in patients with MERS. In contrast to known stimuli (serum osmolality, hemodynamic changes), IL-6^[30]^ may cause vasopressin (antidiuretic hormone) release as a secondary messenger, leading to hyponatremia. During inflammation, several proinflammatory cytokines (IL-6, TNF-α, IL-1) are secreted in the systemic circulation to initiate the so-called “acute phase response.” Cytokines can independently or synergistically stimulate the hypothalamus and pituitary gland and induce vasopressin release, which causes hyponatremia. Hypotonic hyponatremia^[31]^ results in the entry of water into the brain, resulting in cerebral edema. As axons in SCC are tightly packed, it is possible that interstitial edema (water situated between the unmyelinated axons) could have reduced diffusion, which also explains the selective splenial lesions in SCC. In addition, elevation of IL-6 and urinary β2MG reflected an excessive immune response, conforming to the pathophysiology of immune system activation. This also explains why MERS had no direct association with CNS infection or inflammation.

In the case of the 31-year-old man with MERS secondary to viral encephalitis, the clinical presentation was nonspecific, as viral encephalitis may mask the symptoms of MERS. As for the diagnosis of viral encephalitis, both cerebrospinal fluid and cytological classification of serum considered viral infection and antiviral therapy proved effective. In cases of acute or past infection of Epstein–Barr virus, VCA IgG can be present without VCA IgM or EBNA-1 IgG. However, we did not follow up on the changes in the antibody profile, acute infection of Epstein–Barr virus cannot be confirmed. We also discovered that the shape of the lesion on MRI was variable, the SCC lesions were ovoid or round on the fourth day. Subsequently, on the fifth day, lesions were ovoid and irregularly extended into the lateral portion as a “boomerang sign.” The pathogenesis may be attributed to cytotoxic edema caused by hyponatremia and destruction of the blood–brain barrier caused by viral encephalitis. This may increase the sensitivity of the brain cells to hyponatremia. Regrettably, we did not check the patient for IL-6 in the serum or CSF. The patient did not have a severe disturbance of consciousness (such as coma) or other areas of extra callosal lesions. His favorable prognosis (no sequelae) is in accord with the study of Zhang et al.^[3]^ In conclusion, in patients with encephalitis accompanied by hyponatremia, elevated IL-6 or urinary β2MG, and exacerbations such as sudden somnolence, delirium, confusion, and seizures, the possibility of secondary MERS, in addition to the progression of encephalitis, should be considered. Sodium supplementation and immunosuppressive treatments may assist with the treatment of MERS. Whether the level of hyponatremia is related to the severity of MERS requires further research.

## Acknowledgments

The authors would like to thank Editage (www.editage.com) for English language editing.

## Author contributions

**Conceptualization:** Bichuan Shi, Jiao Li, Ji-wei Jiang, Meixin Li, Jian Zhang, Xiu-li Shang.

**Data curation:** Bichuan Shi, Jiao Li, Ji-wei Jiang.

**Formal analysis:** Bichuan Shi, Jian Zhang.

**Funding acquisition:** Xiu-li Shang.

**Investigation:** Bichuan Shi, Jiao Li.

**Methodology:** Bichuan Shi.

**Resources:** Bichuan Shi, Ji-wei Jiang, Meixin Li.

**Software:** Bichuan Shi, Ji-wei Jiang.

**Supervision:** Xiu-li Shang.

**Validation:** Meixin Li, Xiu-li Shang.

**Visualization:** Xiu-li Shang.

**Writing – original draft:** Bichuan Shi.

**Writing – review & editing:** Xiu-li Shang.
